# *Acetobacter indonesiensis* Bacteremia in Child with Metachromatic Leukodystrophy

**DOI:** 10.3201/eid2209.160566

**Published:** 2016-09

**Authors:** Rebekka Kohlmann, Karin Barenberg, Agnes Anders, Sören G. Gatermann

**Affiliations:** Ruhr-Universität Bochum, Bochum, Germany (R. Kohlmann, A. Anders, S.G. Gatermann);; Institute of Medical Laboratory Diagnostics Bochum, Bochum (R. Kohlmann, S.G. Gatermann);; Marienhospital Herne, Herne, Germany (K. Barenberg)

**Keywords:** Acetobacter indonesiensis, acetic acid bacteria, metachromatic leukodystrophy, catheter-related bloodstream infection, bacteria

**To the Editor:** *Acetobacter indonesiensis*, first described in 2000 (*1*), belongs to the group of acetic acid bacteria (AAB), which includes the genera *Acetobacter*, *Gluconobacter*, *Asaia*, *Granulibacter*, and others in the family *Acetobacteriaceae*. AAB are of great industrial interest for use in vinegar fermentation processes because they oxidize alcohols or sugars incompletely, which leads to acetic acid accumulation (*2*). AAB are widespread in nature and can be isolated from various sources, including vinegar, alcoholic beverages, tropical fruits, and flowers (*1*,*2*). AAB have rarely been associated with human disease. We describe a case of *A. indonesiensis* bacteremia in a child in Germany.

A 9-year-old girl with late-infantile metachromatic leukodystrophy was admitted to Marienhospital Herne, Herne, Germany, on February 9, 2015, for elective fundoplication. Because of her advanced neurologic disability, she required extensive nursing care and had several invasive medical devices, including a port catheter (detailed patient data in Technical Appendix). Soon after her admission, fever developed, and C-reactive protein levels increased (Technical Appendix Table 1). Physical examination and further diagnostic investigations (e.g., chest radiograph) revealed no focus of infection. Because she had experienced recurrent pneumonia and candidemia (the latter led to port catheter exchange 1 month previously), antimicrobial drug treatment with piperacillin/tazobactam and caspofungin was administered.

One blood culture, drawn from the port catheter on day 3 of the hospital stay, yielded slowly growing multidrug-resistant bacteria (agar diffusion indicated zones of inhibition only for imipenem, meropenem, fosfomycin, and tigecycline). By partial sequencing of the 16S rRNA gene, we identified the isolate as *A. indonesiensis.* Details of microbiological analyses, colony morphologic features, 16S rDNA-based phylogenetic analysis, and antimicrobial drug susceptibility testing results are given in the online Technical Appendix.

Because the patient clinically responded to piperacillin/tazobactam and caspofungin treatment, therapy was continued for 15 days, although piperacillin/tazobactam showed no in vitro activity against the *A. indonesiensis* isolate. Despite the patient’s improved condition, 1 control blood culture drawn from the port on hospital day 10, while she was receiving antimicrobial treatment, yielded *A. indonesiensis*, although another blood culture drawn peripherally on hospital day 14 yielded no growth. Port catheter exchange was advised but was not performed, according to the parents’ wishes.

The first report of human infection with AAB can be traced to 2004, when peritonitis, associated with *Asaia bogorensis*, was reported in a peritoneal dialysis patient (*3*). Further reports include a description of *Granulibacter bethesdensis* as a cause of lymphadenitis in patients with chronic granulomatous disease (*4*), isolation of *Gluconobacter* spp. from a culture of blood from an intravenous drug user and of *Gluconobacter* spp. and *Asaia* spp. from sputum samples of cystic fibrosis patients (*5*), a case of *A. bogorensis* bacteremia in an intravenous drug user (*6*), and central venous catheter–associated cases of *Asaia lannaensis* bacteremia in a child with cancer who had received a bone marrow transplant (*7*) and in children who had idiopathic dilated cardiomyopathy (*8*).

Regarding *Acetobacter* spp., only 2 reports on human infection have been published: *A. cibinongensis* bacteremia in a patient receiving chronic hemodialysis with signs of an infected arteriovenous fistula and suspected intravenous drug abuse (*9*) and *A. indonesiensis* pneumonia in a cystic fibrosis patient who was receiving immunosuppressive treatment because of a recent lung transplant (*10*). Similar to the case we report, species identification in those cases was achieved only with the help of sequencing methods in both cases. In the *A. indonesiensis* pneumonia case, results of antimicrobial drug susceptibility testing found that the bacteria showed multidrug resistance, as in the case we report, but susceptibility to aminoglycosides.

Of note, the aforementioned AAB infections all occurred in chronically ill patients or intravenous drug users. Similarly, children with metachromatic leukodystrophy are prone to healthcare- and device-associated infections involving opportunistic pathogens, and frequent use of broad-spectrum antibiotics may predispose the children for infection with multidrug-resistant bacteria. In the case we report, frequent accessing of the port, including for parenteral nutrition, may have further promoted microbial colonization.

Because a focus of infection was not apparent and because *A. indonesiensis* grew in 2 blood cultures independently drawn from the port but not in the blood culture obtained from peripheral venipuncture, we assume the patient’s port catheter harbored the infectious agent. The fact that several previously reported AAB infections were catheter-associated may further support our suspicion. However, we could not confirm this assumption because the port was not removed and cultured.

The patient clinically responded to piperacillin/tazobactam and caspofungin treatment, despite a lack of in vitro activity against the *A. indonesiensis* isolate. Although this response might be explained by the presence of a second pathogen (which was not cultured but covered by the given antimicrobial agents), the control blood culture drawn from the port still yielded *A. indonesiensis* and at least argues for persistent colonization of the port. Because of pathogen persistence in blood culture and limited therapeutic options owing to the multidrug-resistance of the isolate, we believe the port should have been removed in this case.

Technical Appendix Supplementary patient data and results of microbiological analyses for case of *Acetobacter indonesiensis* bacteremia in child with metachromatic leukodystrophy.
