# Supplementary material for: Acetobacter indonesiensis Bacteremia in Child with Metachromatic Leukodystrophy
Source: Emerg Infect Dis. 2016 Sep;22(9):1681–3. doi: 10.3201/eid2209.160566 (PMC4994355; doi:10.3201/eid2209.160566)
Supplement: Technical Appendix — Supplementary patient data and results of microbiological analyses for case of Acetobacter indonesiensis bacteremia in child with metachromatic leukodystrophy. [file 16-0566-Techapp-s1.pdf]

Article DOI: http://dx.doi.org/10.3201/eid2209.160566

Acetobacter indonesiensis Bacteremia in Child with Metachromatic Leukodystrophy

Technical Appendix

Supplementary Patient Data

The patient was a 9-year-old child with late-infantile metachromatic leukodystrophy (MLD). MLD is an autosomal recessive, inherited, lysosomal storage disease, caused by deficiency of the enzyme arylsulfatase A (*1–3*). It involves accumulation of sulfatides in the nervous system, leading to progressive degeneration of myelin sheaths. In the most common late-infantile form, affected children exhibit developmental regression at the age of 12–24 months (*2*,*3*). Symptoms include muscle weakness and loss of previously acquired motor skills with progression to spastic tetraplegia, compromised vision and hearing, dysarthria, dysphagia, impaired breathing, incontinence, seizures, and dementia. Thus, MLD leads to progressive neurologic disability and dependency on nursing care, which ends in a decerebrate state and eventually death within 5–10 years after onset of symptoms. In addition, children with MLD may be considered prone to infections. They often die of pneumonia because of impaired breathing and dysphagia/aspiration (*2*,*3*). Also, healthcare- and device-associated infections may evolve while the patient is dependent on nursing care; for example, catheter-related bloodstream infections are common when the patient has intravascular devices (*4*,*5*). Treatment of MLD is limited to management of pain and symptoms (*1*,*3*).

When the patient was admitted to the hospital, she exhibited an advanced state of MLD. She was bedridden, had a tracheostomy tube for intermittent mechanical ventilation support, suctioning and bronchodilator/corticosteroid inhalation therapy, a percutaneous endoscopic gastrostomy feeding tube with a jejunal extension for enteral nutrition, and an implanted port catheter for application of drugs and parenteral nutrition in case of vomiting, which precluded application via the percutaneous endoscopic gastrostomy feeding tube. Because of gastroesophageal reflux and recurrent vomiting, the port catheter was frequently used, and the

patient was admitted for elective fundoplication. She was treated with proton pump inhibitors and antiemetics. In addition, she received muscle relaxants, anticonvulsants, and analgesics because of spasticity and seizures. In the past, she had suffered from recurrent pneumonia, with persistent *Pseudomonas aeruginosa* and *Enterobacteriaceae* colonization of the lung and tracheostomy tube. In the previous 2 months, she had, in addition, experienced recurrent *Candida tropicalis* bloodstream infection, which had been treated with caspofungin and port catheter removal with new port implantation.

As described in the letter, fever and increased C-reactive protein levels developed in the patient on hospital day 3 (Technical Appendix Table 1), most likely because of a port catheter–related bloodstream infection. Further laboratory parameters were normal, apart from a previously known and probably unspecific isolated elevation of gamma-glutamyltransferase (not shown), and physical examination and further diagnostic investigations (e.g., chest x-ray film) revealed no other focus of infection. *Acetobacter indonesiensis* was isolated not only from the blood culture drawn on day 3 from the port but also from the control blood culture drawn on day 10, even though the patient clinically responded to piperacillin/tazobactam and caspofungin treatment (Technical Appendix Table 1). Because of pathogen persistence, a port catheter exchange was recommended, but it was not performed because the patient's discharge was advanced to an earlier time, according to the parents' wish and against medical advice. The initially planned fundoplication was not conducted because of the advanced MLD after discussion of the patient case at the Clinical Ethics Committee.

Results of Microbiological Analyses

The blood culture drawn from the port catheter on day 3 of the hospital stay flagged positive after 72 hours of incubation. Gram stain revealed pleomorphic, mostly gram-negative rods, and subculture at 37°C and 5% CO_2 yielded growth of very small colonies on Columbia sheep blood agar and chocolate agar, but not on MacConkey agar (Technical Appendix Figure 1).

Species identification of the catalase-positive, oxidase-negative bacteria could not be achieved using matrix-assisted laser desorption/ionization (MALDI-TOF) mass spectrometry. Thus, partial sequencing of the 16S rRNA gene was performed by using primers established by

the Bund/Länder-Arbeitsgemeinschaft Gentechnik (LAG), Germany (16S-fw: GAA GAG TTT GAT CAT GGC TCA G; 16S-rev: ACG ACA GCC ATG CAG CAC CT). The sequence of our isolate has been deposited in GenBank (under accession no. KU976968), and it matched those of *A. indonesiensis* isolates deposited in the GenBank database (99.8% identity). 16S rDNA-based phylogeny confirmed affiliation to the species *A. indonesiensis* (Technical Appendix Figure 2).

Antimicrobial susceptibility testing was done by disk diffusion; devices for automated susceptibility testing were not used because of the slow pathogen growth. Zones of inhibition were merely detected for imipenem, meropenem, fosfomycin, and tigecycline (Technical Appendix Table 2). Interpretation of results (susceptible/intermediate/resistant) was not done because breakpoints for *Acetobacter* spp. are not available in the recommendations of the European Committee on Antimicrobial Susceptibility Testing (EUCAST).

References

1. Biffi A, Lucchini G, Rovelli A, Sessa M. Metachromatic leukodystrophy: an overview of current and prospective treatments. Bone Marrow Transplant. 2008;42(Suppl 2):S2–6. PubMed http://dx.doi.org/10.1038/bmt.2008.275

2. Fluharty AL. Arylsulfatase A Deficiency. In: Pagon RA, Adam MP, Ardinger HH, Wallace SE, Amemiya A, Bean LJH, et al., editors. GeneReviews® [Internet]. Seattle (WA): University of Washington, Seattle; 1993-2015.; 2006 May 30 [updated 2014 Feb 6].

3. van Rappard DF, Boelens JJ, Wolf NI. Metachromatic leukodystrophy: Disease spectrum and approaches for treatment. Best Pract Res Clin Endocrinol Metab. 2015;29:261–73. PubMed http://dx.doi.org/10.1016/j.beem.2014.10.001

4. Mermel LA, Allon M, Bouza E, Craven DE, Flynn P, O’Grady NP, et al. Clinical practice guidelines for the diagnosis and management of intravascular catheter-related infection: 2009 Update by the Infectious Diseases Society of America. Clin Infect Dis. 2009;49:1–45. PubMed http://dx.doi.org/10.1086/599376

5. Mirijello A, Impagnatiello M, Zaccone V, Ventura G, Pompa L, Addolorato G, et al.; Internal Medicine Sepsis Study Group. Catheter-related bloodstream infections by opportunistic pathogens in immunocompromised hosts. Eur Rev Med Pharmacol Sci. 2015;19:2440–5. PubMed

6. Flandrois JP, Perrière G, Gouy M. leBIBIQBPP: a set of databases and a webtool for automatic phylogenetic analysis of prokaryotic sequences. BMC Bioinformatics. 2015;16:251. PubMed http://dx.doi.org/10.1186/s12859-015-0692-z

7. Gouy M, Guindon S, Gascuel O. SeaView version 4: A multiplatform graphical user interface for sequence alignment and phylogenetic tree building. Mol Biol Evol. 2010;27:221–4. PubMed http://dx.doi.org/10.1093/molbev/msp259

Technical Appendix Table 1. Selected results of blood laboratory testing and temperature measurement at admission (day 1), at blood culture positivity (days 3 and 10) and a few days before discharge (day 14)*

		Patient's result			
Parameter (unit)	Reference	Day 1	Day 3 (drawn from port)	Day 10 (drawn from port)	Day 14 (drawn peripherally)
Blood culture	Neg.	NA	Pos.	Pos.	Neg.
Body temperature (°C)	<37.0	36.1	39.5 ↑	39.0 ↑	37.3
Leucocytes ($10^3/\mu L$)	4.8–12.0	12.4	5.4	12.6	12.1
C-reactive protein (mg/dL)	<0.5	0.1	30.5 ↑	4.6 ↑	0.5

*N/A, not applicable; neg., negative; pos., positive; ↑, increased.
Pathologic/changed values are marked by arrows.

Technical Appendix Table 2. Results of antimicrobial susceptibility testing done by disk diffusion

Antimicrobial agent	Disk content (μg)	Zone diameter (mm)
Ampicillin	10	6
Piperacillin/tazobactam	30/6	6
Cefotaxime	5	6
Ceftazidime	10	6
Imipenem	10	18
Meropenem	10	12
Ertapenem	10	6
Fosfomycin/glucose 6-phosphate	50/50	19
Gentamicin	10	6
Trimethoprim/sulfamethoxazole	1.25/23.75	6
Tigecycline	15	15
Ciprofloxacin	5	6

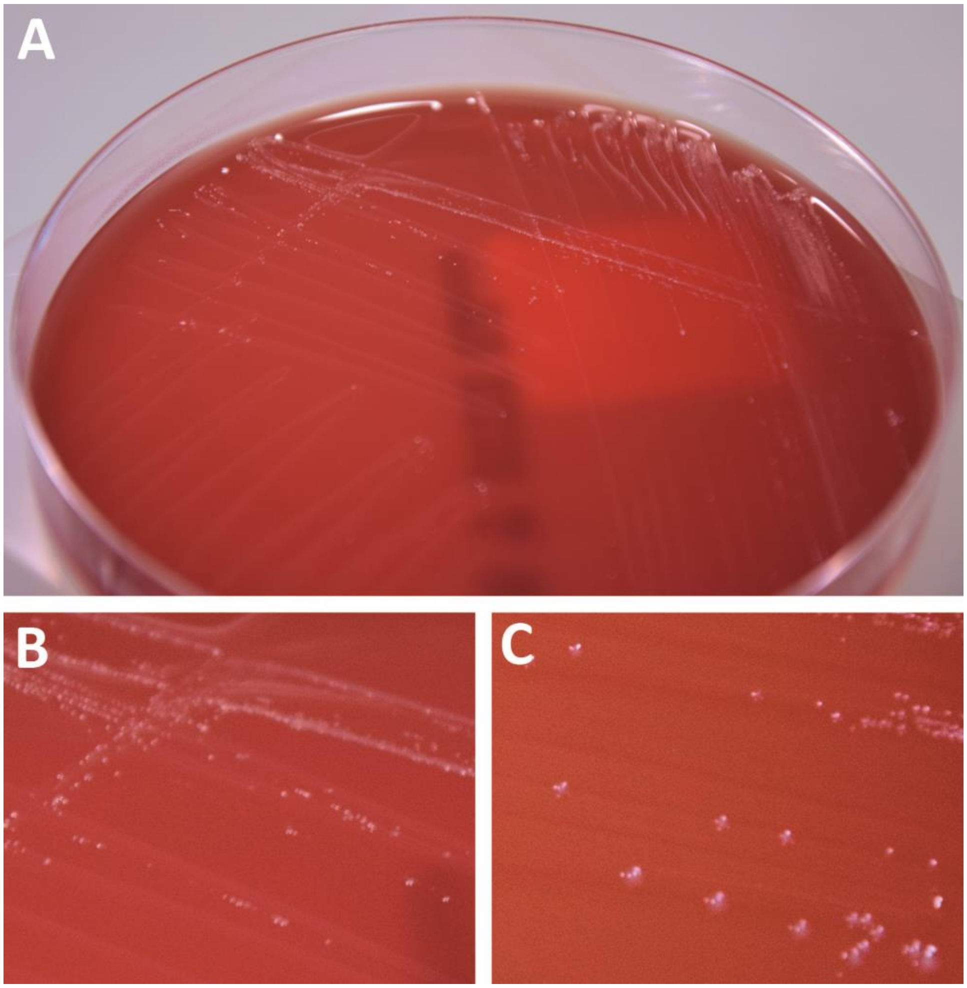

Technical Appendix Figure 1. *Acetobacter indonesiensis* grown on Columbia agar with 5% sheep blood after 48 h incubation (A). For better demonstration of colony morphologic features, we show 2.5-fold enlarged image sections of photos taken after 2 days (B) and 6 days (C).

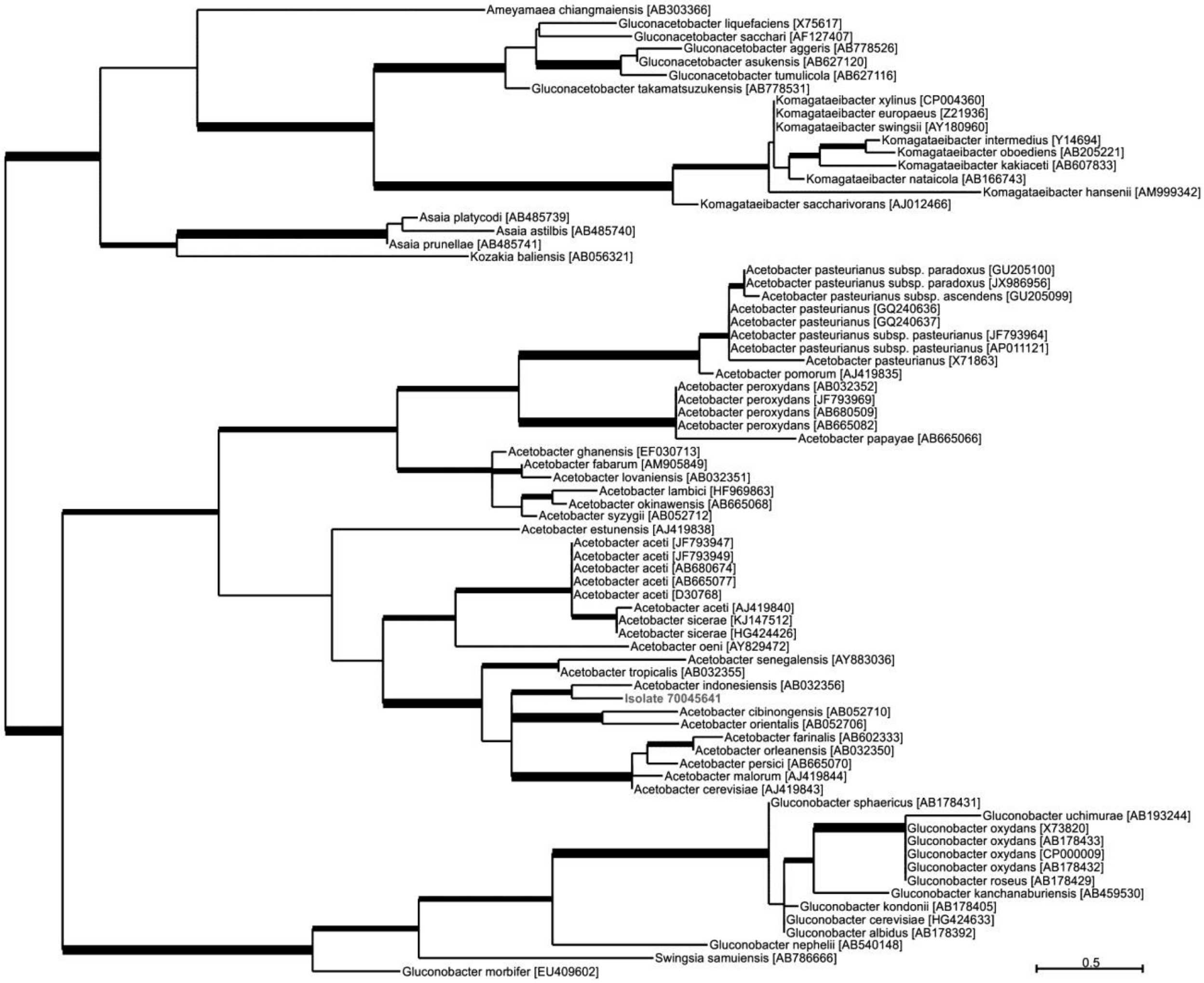

Technical Appendix Figure 2. Phylogenetic tree showing the position of our *Acetobacter indonesiensis* isolate within the genus *Acetobacter* and other genera of acetic acid bacteria. The tree was constructed by using the publicly available leBIBI^{QBPP} program which is based on 16S rDNA comparison by a maximum-likelihood approach (*6*). Our isolate 70045641 is highlighted in red. The 16S rDNA sequences used for comparison were obtained from the GenBank database by using the superstringent algorithm of leBIBI^{QBPP}, and accession numbers are indicated in brackets. The leBIBI^{QBPP} program uses the "branch width as support" option of SEAVIEW (*7*); the support is evaluated by Shimodaira-Hasegawa(SH)-like computation, and the largest width corresponds to SH>0.95 and can be considered as statistically significant, whereas the minimal line width (plain-line) is used when SH ≤ 0.80 and in this case the support is not sufficient. Scale bar indicates nucleotide substitutions per site.
